# Healthcare providers’ perceptions of sexual health concerns among female patients with cervical and breast cancer and survivors in Rwanda: a qualitative interview study

**DOI:** 10.1136/bmjph-2024-002348

**Published:** 2026-03-23

**Authors:** Johannes Espmark, Karin Båge, Dieudonne Rutagumba, Andrea Diane Ndoli, Jean Berchmans Uwimana, Nzeyimana N Innocent, Jean Christophe Rusatira, Anna Kågesten

**Affiliations:** 1 Department of Global Public Health, Karolinska Institute, Stockholm, Sweden; 2 Department of Molecular Medicine and Surgery, Karolinska Institutet, Stockholm, Sweden; 3 Healthy People Rwanda, Kigali, Rwanda; 4 Johns Hopkins Bloomberg School of Public Health, Baltimore, Maryland, USA; 5 Department of Population, Family and Reproductive Health, Johns Hopkins Bloomberg School of Public Health, Baltimore, Maryland, USA

**Keywords:** Health Services Accessibility, Qualitative Research, Sexual Health

## Abstract

**Introduction:**

In Rwanda, breast and cervical cancer are the most common cancers among women of reproductive age, posing significant public health challenges. These cancers do not only account for the highest mortality and morbidity, but also impact women’s sexual health and well-being—often overlooked in cancer care settings. Little is known about healthcare providers’ capacity to counsel patients on sexual health in contexts with limited psychosocial cancer services.

**Methods:**

A qualitative cross-sectional study was conducted to explore healthcare providers’ perceptions, attitudes and knowledge concerning sexual health among female patients with cervical and breast cancer and survivors in two regions of Rwanda. Between January and March 2023, semistructured in-depth interviews were conducted with 15 healthcare providers (medical doctors, nurses, midwives) experienced in treating patients with breast or cervical cancer at four facilities. Interviews were recorded, transcribed verbatim and analysed using content analysis to identify emerging themes related to providers’ perspectives on sexual health counselling.

**Results:**

Two main themes emerged, with the first theme ‘Perceived barriers to sexual healthcare for female cancer patients’ highlighting social and cultural taboos related to sexuality, sexual health knowledge and awareness among both providers and patients, and socio-economic barriers. The second theme, ‘Systemic challenges in delivering comprehensive sexual healthcare’, illustrated health systems barriers such as staff shortage and unclear responsibilities, gendered barriers during counselling and ambiguous sexual health information provided. Sexual and gender-based violence as a barrier to sexual health emerged as a cross-cutting theme***,*** with providers noting different forms of violence faced by patients during cancer therapy.

**Conclusion:**

Findings underscore the pressing need to prioritise women’s sexual health and well-being within cancer care settings in a lower-middle income country context such as Rwanda. Health systems efforts are necessary to enhance the sexual health counselling proficiency of current oncology providers, as well as to train new generations of providers.

**WHAT IS ALREADY KNOWN ON THIS TOPIC**

- Sexual health concerns among female patients with cancer, particularly those with cervical and breast cancer, are often overlooked in cancer care settings. Existing research, primarily from high-income countries, indicates that cancer treatments can significantly impact sexual health, but there is limited evidence from low- and middle-income countries, like Rwanda.

**WHAT THIS STUDY ADDS**

- Findings from this qualitative study revealed that cancer care providers in Rwanda face substantial challenges to address sexual health concerns due to cultural taboos, lack of training and systemic barriers such as staff shortages and unclear responsibilities. Experiences shared by providers also underscore sexual and gender-based violence (SGBV) as a pervasive issue that can exacerbate the sexual health challenges faced by many female patients with cancer.

**HOW THIS STUDY MIGHT AFFECT RESEARCH, PRACTICE OR POLICY**

- This study provides a foundation for future research and interventions aimed at improving sexual health outcomes for female patients with cancer in low-resource settings. Findings underscore the need for targeted training programmes to enhance healthcare providers’ capacity to address sexual health and SGBV among female patients with cancer. Policy efforts should focus on integrating sexual health counselling into cancer care and addressing systemic barriers to improve the overall quality of care.

## Introduction

 Increased incidence of cervical and breast cancers is a growing public health concern in sub-Saharan Africa (SSA)^1^ due to rapid epidemiological and demographic transitions.^2^ While cervical cancer is the form of cancer with the highest mortality in SSA, breast cancer accounts for the highest prevalence and morbidity. Women in SSA usually present with more advanced stages of breast and cervical cancer at diagnosis and suffer more than women in high-income countries.^3^ Although only 10% of women aged 15 and above at risk of cervical cancer live in SSA, 20% of global annual new cases and 24% deaths occur in the region.^4^ A similar pattern can be seen for breast cancer mortality in SSA; it is disproportionately higher than in high-income countries despite lower incidence rates.^3 5^

In Rwanda, cervical cancer is the most common cancer among women of reproductive age and the second most common overall, following breast cancer.^6^ As of 2020, breast cancer incidence in Rwanda was 41 per 100 000, with a mortality of 19.4 per 100 000^7^ and the cervical cancer incidence and mortality was 18.7 per 100 000 versus 12.6 per 100 000.^6^ Lifestyle and demographic changes, such as higher meat consumption and an ageing population, contribute to these trends.^8^

These high incidence and mortality figures underscore a pattern of late-stage presentation, which is driven by multiple interrelated social, economic and health-system factors. First, persistent myths, stigma and limited awareness about cancer symptoms discourage early care-seeking. Second, poverty, transport costs and multiple facility visits create financial and logistical strain.^9^ Third, within the health system, delayed referrals, centralised diagnostic services and limited access to screening programmes further hinder early diagnosis. Fourth, gender norms, fear of disfigurement from treatment (such as mastectomy) and patient–provider communication barriers also discourage timely care.^10^ Finally, environmental and epidemiological factors, such as high human papillomavirus prevalence (34%) and misattribution of early symptoms to benign conditions, compound these challenges and contribute to the late-stage detection of cancer among Rwandan women.^11 12^

Evidence from high-income countries indicates that many women treated for cervical and breast cancer experience a decline in sexual health. This may be due to treatment side effects, including reduced libido, weariness, body image changes, pain from surgery and vaginal dryness from hormonal treatment.^13^,^15^ A meta-analysis of 35 studies from mainly high-income countries found that sexual dysfunction affected over 60% of women with cancer, with nearly 78% experiencing it in gynaecological cancers.^16^ Early cervical cancer symptoms, such as vaginal bleeding and odour, may also affect women’s self-esteem and sexual health, leading to self-isolation.^17^ Yet, women often report lacking information and counselling from healthcare providers on the impact of cancer treatment on sexual dysfunction, with sexual health issues ranking among the top unmet needs after treatment shown by studies in North America.^18^

Recognising the impact of cancer treatment on sexual health, healthcare providers need to actively address these concerns to enhance patient well-being and quality of life (QoL).^19^ This includes providing adequate psychosocial care such as counselling services to screen for and respond to issues related to sexual (dys)functioning, as well as sexual and gender-based violence (SGBV) via trauma-informed and violence-informed approaches.^20^ However, there is a shortage of healthcare providers with both oncology and mental health and psychosocial service training in Rwanda and SSA,^21^ including sexual health knowledge and counselling skills.^22^

In Rwanda, the need for counselling and care that is trauma-informed and violence-informed and gender-sensitive is further shaped by the country’s history. During the 1994 genocide against Tutsi, it is estimated that between 250 000 and 350 000 women and girls were raped, leaving deep psychosocial and reproductive health consequences that continue to affect survivors and subsequent generations. These experiences have contributed to enduring stigma, silence and discomfort around sexual and reproductive health factors that still influence provider–patient interactions today.^23^

While attention to the role of healthcare providers in managing sexual health issues in patients with cancer is growing, most research has been conducted in high-income countries, with evidence from SSA and other low- and middle-income settings lacking.^24^ In response to this gap, this study aimed to investigate healthcare providers’ perceptions, attitudes and knowledge related to the sexual health of female patients with cervical and breast cancer and survivors in Rwanda. Such information is critical to effectively address the psychosocial and sexual health challenges associated with cancer diagnosis in the region.

## Methods

### Study design and setting

We conducted a qualitative study as part of a broader research project called ‘LUBRICATE’, which seeks to understand and reduce SGBV against female patients with cancer and survivors by implementing a novel sexual counselling and therapy model in Rwanda. The project was implemented in 2023 by the non-governmental organisation Healthy People Rwanda (https://hprwanda.org). The LUBRICATE project collected data from health facilities using self-administered online surveys and in-depth interviews with healthcare providers and patients. This study focused on healthcare providers’ perceptions, attitudes and knowledge related to sexual health and counselling of female patients with cancer. Interviews were conducted at four referral hospitals in Rwanda. The study followed the Consolidated Criteria for Reporting Qualitative Studies checklist to ensure comprehensive reporting of qualitative data.^25^

### Participants

The study population included healthcare providers involved in cancer care, including registered nurses, midwives and medical doctors. Participants were recruited from four cancer facilities using purposive sampling,^26^ based on their roles, experience and willingness to participate. Eligible participants had to be directly involved in caring for patients with cervical and breast cancer and speak French or English. The sample size was determined by data saturation, which was reached when no new themes or ideas emerged, and participants began to provide similar responses across interviews.^27^ A total of 15 participants were interviewed.

### Procedures

Data were collected between January and March 2023 using qualitative in-depth interviews. The study team developed a semistructured interview guide (online supplemental appendix 1) with open-ended questions and probes to understand healthcare providers’ perceptions and experiences related to counselling female patients with cancer on issues related to sexual health. Minor changes were made after the first interview to better capture attitudes and views. Additional questions specifically focused on SGBV were also added to the initial draft given the broader focus of the LUBRICATE project.

All interviews were conducted face-to-face by the lead author (JE) in French or English, both official languages of Rwanda, depending on the preference of the respondent. Interviews ranged from 17 to 46 min. Before conducting and recording the interviews, participants read and signed an informed consent form (online supplemental appendix 2) and provided verbal consent. All interviews were audio-recorded and transcribed verbatim, with field notes used to document key aspects from the interviews. The interviewer wrote field notes when necessary to aid in probing and to document non-verbal aspects influencing discussions, such as participants’ mood or body language.

### Data analysis

The transcribed interviews were uploaded to Dedoose V.9.0.17 (SocioCultural Research Consultants, LLC, Los Angeles, California, USA) and analysed using Content analysis (Denzin & Lincoln, 2000). The lead author first read all transcripts multiple times to become familiar with the data and note preliminary ideas. Based on this initial familiarisation, an initial codebook aligned with the research questions was developed and reviewed by coinvestigators and through member checking (see below).

Coding was then conducted iteratively, allowing new codes to be added as they emerged from the data. The lead author coded all transcripts, and a coauthor (KB) double-coded a subset of randomly selected transcripts followed by joint discussion. Any differences in codebook application were resolved through consensus with changes to the codes applied to all transcripts. The codes were further sorted into subcategories and categories to identify emerging themes, as shown in table 1. The first coding round generated six broader themes for the project as a whole, which were reduced to two key themes for the purpose of the present analysis and paper. For all quotes in the results section, participants’ names were anonymised and grammar was modified to better convey responses.

**Table 1:** Example of the path from a meaningful unit to theme

Quote	Condensed meaning unit	Code	Subcategory	Category
*“*Normally, in our culture, as Rwanda, that is a taboo. Things related to sex; it is a taboo. Sometimes the patient is not openly to talk, but if you are closer with the patient, you may be open to talk*.*”	Sexuality is considered taboo in Rwandan culture, hindering patient discussion.	Cultural taboo hindering communication	Cultural taboos hinder sexual discussion between provider and patient.	Patient barriers to sexual healthcare
*“*So, because there is no time to discuss with the patient who has such problem, but I know that problem exist”	The provider is experiencing a lack of time to discuss sexual health.	Provider lacking time	Time and stress hindering providers to provide sexual healthcare.	Impact of resource scarcity on sexual healthcare provision

We used several strategies to enhance the trustworthiness of findings as outlined by Lincoln and Guba.^28^ This included the double-coding of selected transcripts and joint development of the codebook, as well as member checking whereby all participants were invited to review and provide feedback on a summary of the preliminary coding framework. This member-checking process fostered transparency and helped ensure alignment between the researcher’s interpretations and participant perspectives. Incorporating participant feedback also strengthened the credibility of our findings and the participant–researcher dialogue. A total of five participants, a third of all participants, (n=5) volunteered to take part in this process.

### Ethical considerations

The LUBRICATE project received ethical clearance from the Rwanda National Ethics Committee (No.110/RNEC/2022). Respondents were informed that their responses would help assess the ongoing psychotherapy programmes, detailing goals, objectives and data use. The project’s research plan and questionnaire were reviewed and approved by the Institutional Review Boards and Ethics Committees of all collaborating hospitals. Informed consent, both written and oral, was obtained from all participants. Only age, sex and occupation were collected as personal identifying information, and respondent identities remained anonymous in the results. Participants were offered no compensation for their participation.

### Patient and public involvement statement

All participants were invited to participate in member-checks to validate the study’s findings. Findings will be shared through publications, seminars and briefs facilitated by the local networks of the research partner Healthy People Rwanda.

## Results

Table 2 presents the characteristics of the 15 participants, most of whom (n=11) were male and the majority medical doctors (n=9); 4 nurses and 2 midwives were also interviewed. Most participants worked in oncology (n=8) or gynaecological (n=5) departments, and 2 in general surgical. All participants were adults with a mean age of 37 years (SD 32–42).

**Table 2:** Characteristics of providers who participated in in-depth interviews, by sex

	Male (n=11)	Female (n=4)	Total (N=15)
Age (mean, SD)	36.45±1.91	39±2.5	37.13±1.65
Department/service (n, %)			
Oncology	5 (45, 45)	3 (75)	8 (72, 72)
Gynaecology	4 (36, 36)	1 (25)	5 (33, 33)
General surgery	2 (18, 18)	0 (0)	2 (13, 33)

Two main themes emerged from our analysis: (1) *perceived barriers to sexual healthcare for female patients with cancer* and (2) *systemic challenges in delivering comprehensive sexual healthcare*. These themes were further made up by three categories and eight subcategories as described in table 3. SGBV emerged as a cross-cutting issue throughout the analysis and is discussed in relation to the themes. Below we present key findings for each theme, category and subcategory, using illustrative and anonymised quotes.

**Table 3:** Themes, categories and subcategories

Themes	Categories	Subcategories
1. Perceived barriers to sexual healthcare for female patients with cancer**.**	1.1 Patient barriers to sexual healthcare. 1.2 Patient awareness and knowledge barriers in accessing sexual healthcare.	1.1.1 Cultural taboos hinder sexual discussion between provider and patient. 1.1.2 Gender dynamics in communication. 1.1.3 Indirect communication from patients. 1.2.1 Patients lack money to finance sexual healthcare. 1.2.2 Patients’ knowledge and awareness affect providers’ capacity to counsel.
2. Systemic challenges in delivering comprehensive sexual healthcare.	2.1 Limited organisation of healthcare personnel hinders capacity for adequate sexual healthcare.	2.1.1 Unclear provider responsibilities within the healthcare system. 2.1.2 Ambiguity in information delivery to patients. 2.1.3 Female patients at unease with male providers.
	2.2 Impact of resource scarcity on sexual healthcare provision.	2.2.1 Time and stress hindering providers to provide sexual healthcare. 2.2.2 Lacking adequate resources and logistics for sexual healthcare.
Sexual and gender-based violence shaping care provision.	Patients exposed to sexual and gender-based violence. Providers lacking resources to manage sexual and gender-based violence.	

### Perceived barriers to sexual healthcare for female patients with cancer

During the interviews, providers described how female patients with cancer within their healthcare setting tend to be reluctant to talk about their sexual health needs or seek related care due to cultural barriers, limited knowledge and awareness, as well as their socio-economic status.

First, broader *social and cultural taboos related to sexuality* were perceived as providers to be key barriers for female patients with cancer to talk about and seek care for their sexual health and well-being. Several providers described how prevailing social and cultural taboos related to female sexuality affect both their own ability, and that of their female patients with cancer, to openly discuss intimate issues such as sexual dysfunction. One provider illustrated this by discussing how female patients might hide sexual health issues from their providers because of the perceived stigmatisation and social taboo to discuss issues, including exposure to SGBV. As reflected in the quote from a male midwife, participants emphasised the need to build trust between patient and provider to overcome such taboo:

Normally, in our culture, as Rwanda, that is taboo. Things related to sex; it is a taboo. Sometimes the patient is not open to talking, but if you are closer to the patient, you may be open to talking. But, for us as care providers, we are comfortable to talk about sexual and gender-based violence, but the problem is that on the patients, where she is not comfortable to talk openly. (Nurse, male)

The providers’ narratives further suggested that cultural factors may play a significant role in shaping the taboo related to talking openly about sexual health within the context of cancer care. One provider exemplified how patients who seek consultation may initially raise unrelated questions rather than directly addressing their sexual problems. In contrast, a female gynaecologist expressed that sexual taboo among patients is not really a concern, but that the problem lies among her male colleagues not being open enough to discuss such matters during counselling.

The healthcare providers further emphasised that their patients lack *knowledge and awareness about sexual health* in general and have little awareness of where and how to seek preventive or medical care for problems such as sexual dysfunction. Other providers, such as a male oncologist, argued that insufficient information to patients concerning sexual health-related side effects of treatments might result in a lack of awareness regarding the necessity of seeking medical attention for such issues, as well as their right to do so.

It is not only on the clinicians’ side but in general […] there is little awareness [about sexual health], right? There is little awareness around that, and that involves people seeking [care]. [Both from] people providing care and people seeking care. So, it is multiple aspects. (Oncologist, male)

In addition, providers noted how the general lack of awareness about cancer led patients to present to clinics with severe symptoms typical of advanced stages of breast and cervical cancer. As noted by one respondent:

The community doesn’t know […] when to report to the health settings, when to go to the doctor, when to go for another healthcare provider. Awareness, we need to raise awareness in our community for oncology cases […] in general. Because we meet people with advanced stage and people did not know the warning signs of cervical or other breast cancers. (Midwife, male)

As a result, providers described it as especially challenging to prioritise their patients’ sexual health due to their late-stage disease and unfavourable prognosis on presenting at the clinic.

Furthermore, participants described *socio-economic barriers* whereby the financial burden of cancer care, including the cost of treatment, medications, lubricants and travel expenses to healthcare facilities, impedes female patients with cancer and survivors from seeking and receiving adequate care, including sexual healthcare. Many providers noted their patients often have to pay out of pocket to travel to different healthcare facilities for treatment, which may not be possible. They also described how national health insurance does not cover pharmaceuticals relevant to enhancing sexual health, such as lubricants:

For the lubricant, this is the issue because for women there is not support, they need it to buy it, that is the issue. (Midwife, female)

Problems related to affordability issues with breast prosthetics for patients were also given as examples.

### Systemic challenges in delivering comprehensive sexual healthcare

The second main theme relates to healthcare system limitations in counselling, responsibility and continuity of care, creating inconsistencies in managing cancer treatment side effects. Participants highlighted how the *limited organisation of healthcare personnel hinders capacity for adequate sexual healthcare*, including unclear responsibilities and lack of time for counselling, leaving providers uncertain and hindering continuity of care.

A lack of counselling and enough counsellors. […] We don’t have that […] particular protocol for it. Everyone does it their way. So, there’s no particular protocol to say that if a patient comes at their first visit, we have to proceed like this. So that’s a challenge. (Oncologist, female)

Providers also described an *ambiguity in the (sexual health) information delivered to patients,* citing how they are not able to offer advanced care or provide information on side effects to patients with cancer at their clinic because they routinely referred such patients to specialised cancer centres. One gynaecologist suggested that having the same individual provider manage a patient’s care could simplify treatment and allow more time to counsel and explain side effects.

I think it’s because if […] I’m the one to manage the patient, I’ll be having more time with them, explaining their disease, progression, management, and possible complications. And even complications of the disease, also for complications of the management because if they go for chemoradiation, of course, there will be some complications. (Gynaecologist, male)

*Gendered barriers within the health system* also emerged, as many male providers found it difficult to counsel female patients on sexual health due to the social and cultural taboos described above. A female oncologist expressed that female patients with cancer usually feel more at ease to openly talk about sexual health issues with healthcare providers of the same sex.

I’ve met some of our patients. Like they’ve already dealt with a colleague, a male colleague of mine. And there are certain things that were not comfortable to talk about with the men. And maybe it depends on the person they meet. (Oncologist, female)

She further noted that some of her female patients with cancer had not been informed by her male colleagues about certain aspects of their condition, such as stenosis and sexual intercourse, highlighting the gender disparity in communication and the need for improved communication. One male surgeon concurred and mentioned that female patients with cancer sometimes request a female provider, noting that this might be due to discomfort and lack of trust towards males due to experiences of violence and abuse.

About one or two weeks ago patient explicitly requested to be reviewed with a female doctor. So, yes. I feel that we still also have that problem, and we don’t know how to truly put them at ease. Because they might, most likely, I think, be abused by males. And, again, to meet with a male doctor, is not that easy[…] (General surgeon, male)

Participants further discussed the *impact of health systems resource scarcity on sexual healthcare provision*, including staff shortages leading to stress and lack of time for existing staff to provide quality care. One provider mentioned seeing up to 20 patients with cancer a day, noting that to devote enough time to counselling, he would only be able to see about three patients. Understaffing and a high patient influx further prevented providers from allocating sufficient time to address sexual health issues effectively:

So, the main challenge is that we have a lot of patients that are waiting. And sometimes, as doctors, we don’t have the time that is required for such counselling. So, I can say that we are not doing good counselling because of the time, that we don’t have […] because good counselling takes time. That is the main challenge. (Gynaecologist, male)

Some participants further found the facilities where care is given to be substandard and lacking the privacy needed to discuss intimate matters related to sexual health. They highlighted the need for private rooms where patients can receive sexual counselling without being disturbed by other patients or healthcare workers. As noted by a male midwife, doing so might help patients feel more at ease and encourage them to openly discuss psychosocial issues related to their cancer diagnosis.

We don’t have a specific room for cancer. […] They have a severe disease which other people think is a big matter, and everyone can be fearful. These patients need a specific room or safe room where they can express open-ended […]. (Midwife, male)

Lastly, many providers reported missing the necessary resources, such as lubricants and vaginal dilators, to address sexual health issues in this patient group. They emphasised the importance of having these materials and expressed challenges in providing comprehensive care and meeting the needs of their patients effectively due to their scarcity.

There is lack of also logistics that I have told you about like, lubricants and the other, you know, at artificial penis [vaginal dilators]. But also, we need a very comfortable environment that we don’t have […] (Oncologist, male)

### Sexual and gender-based violence shaping care provision

A cross-cutting issue encountered in all interviews was *SGBV* as something inherent to the lives of many female patients with cancer, affecting both their ability to seek care and their broader (sexual) health and well-being. Several providers expressed how many of their patients are exposed to SGBV after a cancer diagnosis. Such violence could take different forms, including physical, sexual and economic violence, as reflected in the quote below where a provider described how a female patient’s partner did not want to pay for her cancer treatment.

Sometimes they don’t tell you about it clearly, but they told you, He can’t pay my […] He doesn’t pay me, or he can’t […] help me to pay […] treatment. (Nurse, female)

Several providers described witnessing their female patients being left by their partners following diagnosis. One reported how a husband exhibited rejection towards the patient, perceiving her as ‘no longer a woman’ and separating her from her children. Another provider reported instances where certain patients were abandoned by their entire families following diagnosis, placing them in challenging social circumstances. Some providers reported patients being exposed to rape or other forms of sexual violence because of their diagnosis.

I remember this case it was breast cancer, double breast cancer. She was on treatment; she was on chemo, and the husband would maybe wait for her two days post-chemotherapy and go ahead. And it felt like rape, and the woman latterly just came with her husband and told me, ‘Tell him that is bad, tell him’ You know, it was very poor. I do not think there was a lot of consent to that (Oncologist, male)

While a significant number of participants associated cancer therapy with heightened vulnerability to SGBV, they also displayed varying attitudes towards SGBV including their role in addressing it. Only one provider acknowledged actively addressing these concerns within the clinical follow-up, whereas another provider remarked that it fell outside the physician’s responsibility. Providers further expressed that it is challenging to help patients who disclose SGBV, having limited options to follow-up and provide adequate support.

It is difficult. As a doctor, it is like the gender-based violence I was talking about. So, then you are allowed to report it to the authorities, but eventually, the investigations are made by the police […], And we don’t usually have a true follow-up on those cases. (Surgeon, male)

Some providers also mentioned couple counselling as a potential strategy to facilitate the relationship between the two in hopes of lowering the risk for miscommunication and risk for SGBV.

## Discussion

We explored healthcare providers’ perceptions, attitudes and knowledge related to the sexual health of female patients with cervical and breast cancer and survivors in Rwanda. Providers noted that female patients with cancer in this context typically do not seek help for sexual health concerns. We identified various obstacles that impede effective service delivery to meet women’s sexual health needs postdiagnosis or treatment. Below, we discuss key findings and provide recommendations for future research and programmes.

First, our finding that providers face challenges in sexual health counselling of female patients with cancer, primarily due to the shortage of time and personnel, confirms the limited available research on the topic in Rwanda. A study on cancer control in Rwanda found inadequate human resources to be a significant barrier to cancer care, highlighting the need to recruit and train more oncologists and staff, including onco-pharmacists, pharmacy technicians and oncology nurses.^8^ Similarly, a 2023 systematic review on cancer care in SSA highlighted how human health resource constraints, including demotivated and burnt-out staff, and inadequate specialist training are key barriers to quality cancer care in the region.^29^

However, we also found additional health system barriers related to inadequate continuity of cancer care, where patients must seek consultations from multiple providers and hospitals. Previous literature^30^ and participants’ narratives revealed that developing a rapport with one’s healthcare provider is crucial for patients to feel comfortable discussing intimate topics like sexual health. Nonetheless, our research suggests that establishing this level of trust is challenging in Rwanda, as patients with cancer routinely engage with different providers, each allocated limited time for individual patients.

We further found that oncology staff prioritised treating more patients with cancer over specific psychosocial counselling related to sexual health. This aligns with Rwanda’s commitment to the Right to Health under the International Covenant on Economic, Social and Cultural Rights.^31^ Progressive realisation of the right to health in the context of cancer means that resources will first be devoted to general cancer care and saving lives. As cancer care expands, more efforts can ensure holistic, high-quality care, including sexual health counselling.^32^ While extensive sexual health counselling might be hard to provide given Rwanda’s current healthcare system, as it develops, resources should tackle barriers to sexual healthcare to ensure increased accessibility.

Beyond health systems and resource constraints, gendered social and cultural taboos related to (female) sexuality stood out as key barriers to effective counselling, with providers noting patients’ reluctance to disclose and talk about sexual health problems. This aligns with how taboos and stigma related to female sexuality hinder access to general sexual and reproductive health services in Rwanda,^33^ as in broader SSA.^34^ Our study supports these findings indicating that similar taboos might impede the provision of sexual health counselling and care to female patients with cancer. Potential reasons for this cultural taboo identified in the literature include inadequate sex education among Rwandan youth and the lack of cross-generational conversations about sexuality.^35^ The insufficient availability of accurate information on sexuality may also have contributed to the recurring narrative shared by several study participants regarding the limited awareness of sexual health among female patients with cancer. Previous research highlights the importance of providers initiating conversations about sexual health when patients are hesitant to discuss these issues.^36^ Quality improvement efforts in contexts like Rwanda are necessary for providers to fulfil this role effectively.

Taboos related to sexual health may further underpin the gendered health systems challenges in cross-gender counselling, with male providers reporting discomfort when discussing sexual health with female patients, and female providers expressing concerns about the counselling skills of their male counterparts. Research from high-income countries like the USA shows male providers face more difficulty in taking sexual histories from female patients than their female counterparts.^37^ Given the gender distribution among healthcare providers in Rwanda—with approximately 87% of practising physicians being male,^38^ it is imperative to enhance sexual health training of male providers, as well as to increase the number of female providers. Such efforts can also involve task sharing by involving trained female nurses and midwives in sexual health counselling as part of cancer care.

Our findings show how providers experienced SGBV as a cross-cutting issue affecting patients’ care-seeking behaviour, QoL, providers’ capacity to counsel patients and SGBV management due to limited healthcare resources. Providers shared stories of female patients being abandoned and/or harassed by partners following cancer diagnosis and experiencing economic violence, leaving them unable to pay for their treatment. This suggests that cancer treatment may aggravate existing gender inequalities and power imbalances,^39^ manifesting via increased violence that the healthcare system can help detect as part of holistic cancer care. While there is limited research on the link between SGBV and cancer in SSA, findings from qualitative studies in the USA indicate that cancer diagnosis may escalate or alter the nature of violence for SGBV survivors.^40^ In Rwanda, an estimated 41.5% of women experience physical or sexual violence in their lifetime.^41^ Yet, providers expressed limited capacity to assist these patients, and some did not perceive SGBV as a problem. Providers and policymakers in Rwanda and elsewhere need to consider how a cervical or breast cancer diagnosis impacts women’s social circumstances, such as being abandoned by their partner or being abused/harassed, and how that might affect their quality of care.

Finally, out-of-pocket payments for cancer care in Rwanda stood out as a key barrier,^8^ with patients paying for both life-saving treatments (like chemotherapy) and life-enhancing treatments (like lubricants).^8^ Providers noted that these expenses made it difficult to address sexual health issues, especially when women are not in control of financial resources. For instance, prescribing lubricants may not guarantee access if the patient bears the financial burden or lacks control over how family money is spent. Research in SSA shows that lack of essential resources in cancer care leads to high prices and catastrophic out-of-pocket spending.^29^ To improve access to cancer-related healthcare services, like sexual health counselling, increasing subsidies for various aspects of cancer care is necessary. This aligns with Rubagumya,^8^ who stressed including all aspects of cancer care (from screening to follow-up) in Rwanda’s community-based insurance scheme.

### Strengths and limitations

A key strength of this study is that it addresses a topic with little research available in low- and middle-income countries, providing evidence to improve the QoL of patients with cervical and breast cancer and survivors. It is among the pioneering studies to explore provider perspectives on sexual healthcare for patients with cancer in SSA and the first of its kind in Rwanda. Despite a small sample size (15 healthcare providers), data saturation was achieved, strengthening the credibility of our findings. The low number of female participants likely reflects Rwanda’s skewed health system gender distribution. Given the qualitative design, findings cannot be generalised to all Rwandan cancer care providers. The sampling from different hospitals in both urban and rural settings nonetheless helped to enhance the transferability of the findings. It is also possible that the fact that interviews were done in English or French, rather than the local language Kinyarwanda, led to misinterpretations; however, member checks were undertaken with participants to minimise potential data inaccuracies.

### Recommendations

Based on this research, we make the following four recommendations.

1. Strategies are needed to strengthen the oncology workforce and clinic structures to allow time for counselling on sexual health, considering human resource shortages and high patient volumes. Activities could include in-service training for nurses and physicians working in oncology, protected consultation time for psychosocial and sexual health concerns, and measures to improve continuity. These interventions should be piloted and evaluated with patient feedback before wider implementation.
2. Comprehensive sexual health training needs to be prioritised for cancer care providers, with a focus on male provider competence to proactively and respectfully discuss sexual health with female patients. Incorporating sexual health into medical education and training is essential to empower healthcare providers to lead discussions on this topic.^42^ Additionally, policies should prioritise actions to ensure a gender balance among healthcare providers as female patients feel more comfortable discussing intimate details with female providers.
3. Standardised aid sexual health counselling should be developed, such as a QoL screening questionnaire to standardise and assist provider–patient consultations. While such tools would not address the broader structural barriers, implementing QoL questionnaires could serve as a cost and time-effective intervention to enhance the provision of sexual healthcare in Rwanda, as research in Europe has shown that to be effective.^43^
4. Programmes and resources need to be allocated towards SGBV screening and response among female patients with cancer and survivors. Given the high prevalence of SGBV and its profound impact on care-seeking behaviour of female patients with cancer and overall treatment experience, healthcare providers and the broader health system must be equipped with the skills and tools to detect and respond to these issues. Such comprehensive training could enhance providers’ ability to counsel on sexual health and address SGBV. This responsibility does not have to fall solely on oncology specialists; task-sharing with other trained professionals can ensure patients receive support. Additionally, improving the availability of resources and support systems for those experiencing SGBV is crucial to ensure holistic cancer care.

## Conclusion

Several barriers, including social and cultural taboos, staff shortages and lack of resources, limit providers’ perceived capacity to counsel female patients with cancer on sexual health and well-being in Rwanda. Health systems efforts are needed to strengthen the workforce and ensure adequate training of cancer care providers to efficiently detect and respond to sexual health concerns, including exposure to gender-based violence, which may be exacerbated during cancer treatment. Our findings can help guide interventions and research to improve sexual health counselling for patients with breast and cervical cancer in Rwanda, including tools for providers to better adapt to their operating environment. Future qualitative research with women and their partners is needed to understand the impact of cancer on women’s sexual health and exposure to violence, and how healthcare providers can address these concerns.

## Supplementary material

10.1136/bmjph-2024-002348 online supplemental appendix 1

## Data Availability

Data are available upon reasonable request.
