# Supplementary material for: Healthcare providers’ perceptions of sexual health concerns among female patients with cervical and breast cancer and survivors in Rwanda: a qualitative interview study
Source: BMJ Public Health. 2026 Mar 23;4(1):e002348. doi: 10.1136/bmjph-2024-002348 (PMC13034309; doi:10.1136/bmjph-2024-002348)
Supplement: online supplemental appendix 1 [file bmjph-4-1-s001.pdf]

Appendix

Appendix 1. LUBRICATE QUALITATIVE INTERVIEW GUIDE

**Purpose of the Questions**	**Follow up Questions.**
A qualitative survey for healthcare providers	
Screening questions	1. Age:<br>2. Health Facility:<br>3. Department/Service:<br>4. Specialty:<br>5. Gender:<br>6. Are you a part of the LUBRICATE-project with dr. Diane:
1. How do you describe your experience counselling breast and cervical patients from school to when you are practicing as a fully-fledged healthcare provider?	1. Did you receive any formal or informal training about sexual and gender-based violence topics and/or sexual health?<br>2. Have you ever found yourself not able to help a woman cancer patient/ survivor disclosing to you that they want sexual health counselling?<br>3. If you had a sexual health counselling tool for breast and cervical patients accessible, do you think would this be useful to you?<br>4. In regard to your affiliated clinic needs and challenges, what are the tools needed for such counselling tool according to you?<br>5. What are your thoughts on importance of addressing sexual health in relation to the overall health and well-being of women diagnosed with:<br>Breast cancer?<br>Cervical cancer?<br>6. Are you comfortable discussing issues of sexual health and Sexual and gender-based violence with your patients?
2. In general, how do you describe the sexual quality of life of breast and cervical patients and survivors?	1. Have you ever had a woman cancer patient disclosing to you poor sexual quality of life? What did she disclose exactly and how did she do that?<br>2. How did you manage this case?<br>3. Do you know any vaginal lubricants?<br>4. How are lubricants accessible at your affiliated clinic?<br>5. Did you ever needed to prescribe a lubricant for couples or individuals?<br>6. What are the challenges faced by you at your affiliated clinic when you try to provide sexual health counselling and therapy for breast and cervical patients/ survivors? How do you respond to these challenges?

3. How do you do follow up on breast and cervical patients if they ever come to your affiliated clinic?	1. What is the follow up plan of your breast and cervical patients and survivors at your clinic?<br>2. How do you provide counselling to breast and cervical patients and survivors at your clinic?<br>3. At your affiliated clinic, is there a screening tool in use to detect sexual and gender-based violence among patients?<br>4. What information regarding potential side effects from breast and cervical cancer treatments is provided to patients, regarding sexual health?<br>5. What kind of care is offered to breast and cervical cancer patients regarding potential side effects?
4. How do you describe relationship challenges faced by breast and cervical patients on treatments?	1. Have you ever had a woman cancer patient disclosing to you exposure of sexual and gender-based violence? What did she disclose and how did she do that?<br>2. How did you manage that case?
5. Influence of Lubricate project	1. How has project Lubricate contributed to your care to cancer patient?<br>2. How has project Lubricate contributed to the way you address sexual and gender-based violence?<br>3. What percentage do you think will benefit from Lubricate project?
	THANK FOR YOUR COLLABORATION

Appendix 2. Consent form

HEALTHY PEOPLE RWANDA (HPR)

Reg.013/NGO/RGB/2016

Welfare-Safety-Equity

KN 5Rd Street, RN3, Remera, Kigali, Rwanda

Healthy People Rwanda

CONSENT TO PARTICIPATE IN LUBRICATE RESEARCH PROJECT.

I. *Title of Study:* LUBRICATE " transforming sexual health counseling and therapy for women cancer patients and cancer survivors in Rwanda: monitoring, evaluation, learning and research (MELR) Project.

II. Investigators:

Name: Dr. Diane Ndoli Andrea, MD, MMED, Oncologist (Principal Investigator)
Name: Dr. Jean Berchmans UWIMANA, MD (Co-Principal Investigator)

III. Introduction:

You are being asked to be in a research study, collecting data about LUBRICATE " transforming sexual health counseling and therapy for women cancer patients and cancer survivors in Rwanda: monitoring, evaluation, learning and research (MELR) Project. You were selected as a possible participant because we think you have a relationship with this research project. We ask that you read this form and ask any questions you may have before agreeing to be in the study.

IV. Purpose of Study:

- Understanding the current prevalence of different forms of sexual and gender-based violence among women's cancer patients and survivors.
- Demonstrating the current knowledge, counseling, and practices to address adverse effects of cancer treatment on sexuality: vaginal dryness, vaginal stenosis, dyspareunia, and decreased libido.
- Illustrating the current stigma and Sexual Gender-Based Violence experiences of cancer patients and survivors on the ground of adverse effects of the cancer treatments.
- Investigating the current psycho-oncology knowledge and care available for women's cancer patients and survivors.
- Revealing patients' satisfaction, fears, and desires regarding sexual pleasure and sexual health among women's cancer patients and survivors.
- Evaluating the success factors for LUBRICATE model as a tool to address SGBV and improve the sexual health of women's cancer patients and survivors.

V. Risks/Discomforts of Being in this Study:

If you agree to this consent, you have decided that: A team working on LUBRICATE data collection process interrogates you and records your responses. You have also agreed to provide information about the concerns of the above-described LUBRICATE project. There are no other reasonable foreseeable (or expected) risks. There may be unknown risks.

Rwanda National Ethics Committee
Approval Date: 21/04/2022
Expiration Date: 20/04/2023

CS CamScanner

VI. Confidentiality:

The records of this study will be kept strictly confidential. Research records will be held in a locked file, and all electronic information will be coded and secured using a password-protected file. They will be accessed only by the research team and analysts. They will be erased upon completion of the study report.

VII. Payments:

There will be no compensation for participating in the study.

VIII. Right to Refuse or Withdraw:

The decision to participate in this study is entirely up to the participant. You may refuse to take part in the study at any time without affecting your relationship with the investigators of this study. You have the right not to answer any single question and, as well as to withdraw entirely from the interview at any point during the process; additionally, you have the right to request the interviewer not to use any of your interview material.

IX. Right to Ask Questions and Report Concerns:

- You have the right to ask questions about this research study and to have those questions answered by me before, during, or after the research. If you have any further questions about the survey, at any time, feel free to contact:

1. Dr. Diane NDOLI Andrea at andreadiane.ndoli@hprwanda.org /telephone at 0788856738
2. Dr. J. Berchmans UWIMANA at programs@hprwanda.org / telephone +250785545088.

- Should at anytime you have concerns or a complaint to make about the study or research team; you have the right to file a complaint with the following:

o Dr. MAZARATI Jean Baptiste, Chairperson of RNEC by telephone at 0788309807
o Dr. David TUMUSIIME, secretary of RNEC, by telephone at 0788749398

X. Consent:

Your signature below indicates that you have decided to volunteer as a research participant for this study and that you have read and understood the information provided above. You will be given a signed and dated copy of this form to keep, along with any other printed materials deemed necessary by the study researchers.

Subject's Individual Code, signature, and Date:

Investigator's Signature and Date:

Rwanda National Ethics Committee
Approval Date: 21/04/2022
Expiration Date: 20/04/2023
